# The Combined Effects of Iron Excess in the Diet and Chromium(III) Supplementation on the Iron and Chromium Status in Female Rats

**DOI:** 10.1007/s12011-017-1203-z

**Published:** 2017-11-21

**Authors:** Halina Staniek, Rafał W. Wójciak

**Affiliations:** 1 Institute of Human Nutrition and Dietetics, Department of Bromatology and Food Toxicology, Poznań University of Life Sciences, ul. Wojska Polskiego 31, 60-624 Poznań, Poland; 2 Department of Clinical Psychology, Poznań University of Medical Sciences, ul. Bukowska 70, 60-812 Poznań, Poland

**Keywords:** Chromium(III) propionate complex, Fe excess, Iron, Chromium, Rats

## Abstract

Inadequate iron supply has significant consequences to health. There are some relations between the metabolism of different trace elements, such as iron, zinc, copper and chromium. However, the direction of these interactions can be antagonistic or synergistic, and it depends on many factors. The aim of the study was to evaluate the combined effects of supplementary of chromium(III) propionate complex (Cr3) with iron excess on the Cr and Fe status in healthy female rats. The 36 healthy female Wistar rats were divided into six experimental groups (six animals in each) with different Fe levels—adequate (45 mg kg^−1^—100% RDA) and high (excessive—180 mg kg^−1^—400% RDA). At the same time, they were supplemented with Cr(III) at doses of 1, 50 and 500 mg kg^−1^ of diet: C1—control (Fe 45 mg kg^−1^, Cr 1 mg kg^−1^); C50 (Fe 45 mg kg^−1^, Cr 50 mg kg^−1^); C500 (Fe 45 mg kg^−1^, Cr 500 mg kg^−1^); H1 (Fe 180 mg kg^−1^, Cr 1 mg kg^−1^); H50 (Fe 180 mg kg^−1^, Cr 50 mg kg^−1^); H500 (Fe 180 mg kg^−1^, Cr 500 mg kg^−1^). The serum iron level and total iron binding capacity (TIBC) were measured with colorimetric methods. The serum ferritin level was measured by means of electrochemiluminescence immunoassay. The serum transferrin level was measured with the ELISA method. Haematological measurements were made with an automated blood analyser. The Cr and Fe tissular levels were measured with the AAS method. The exposure to a high level of Fe(III) alone or in combination with Cr caused Fe accumulation in tissues, especially in the liver and kidneys, but there were no significant changes in the TIBC, transferrin, ferritin concentration in the serum and most haematological parameters. Moreover, the serum, hepatic and renal Cr concentrations decreased. The doses of supplementary Cr(III) given separately or in combination with high level of Fe(III) disturbed the Cr content in the liver and kidneys of healthy female rats. However, they did not change most of the parameters of Fe metabolism, except the Fe kidney concentration. Supplementary Cr3 decreased the renal Fe level in groups with adequate Fe content in the diet. However, the renal Fe levels increased along with a higher Cr level in the diet in groups with high Fe content. The findings proved a relationship between Fe(III) and Cr(III) metabolism in healthy female rats. However, the direction of change varied and depended on relative amounts of these elements in the diet.

## Introduction

Although iron deficiency anaemia is the most common nutritional deficiency worldwide, the health effects of iron excess deserve special attention [1]. Public health interventions, such as fortification and enrichment of foods with Fe, were undertaken to reduce the prevalence of iron deficiency anaemia and to improve health. These measures as well as Fe supplementation arouse controversy, because additional exposure to dietary iron increases the risk of iron excess [1]. Recently, the health effects of iron excess have received increased attention. Excessive iron intake might be related with the aetiology of some chronic diseases, including diabetes [2], cancer [3], and cardiovascular disease [1, 4].

It is believed that the accumulation or deficiency of trace elements in the human body is often caused by environmental pollution, improper diet, or metabolic disorders [5]. Food fortification and dietary supplementation with pharmaceutical preparations are methods preventing deficiency and alleviate its symptoms. Currently, the market offers a wide range of dietary supplements rich in minerals. However, inappropriate use and dosage may cause excess of some elements and competitive interactions with other minerals.

The results of some studies support the suggestion that the absorption of iron from supplements may be inhibited by other mineral ingredients, such as Ca, Mg, Zn, Cu, and Cr [1]. The biological effects of high iron intake by otherwise healthy individuals are not fully understood. Potential synergistic effects of the iron status and Cr supplementation have received very little attention. High iron intake is most likely the result of dietary supplementation and the consumption of foods fortified or enriched with iron. If Fe absorption is strictly regulated through feedback, modest levels of supplemental iron are unlikely to pose a health risk to otherwise healthy adults [1]. Iron can be harmful when it accumulates in the body. Some people do not eat enough food containing iron to support their health optimally while others have so much Fe that it threatens their health.

It is known that Fe is an essential trace element for human growth and health. It is engaged in many of critical processes [6]. The bioavailability and absorption of this element from the diet are depended by the type and amount of Fe in food, by the presence of promoting and inhibiting factors of Fe absorption in the diet and by one’s individual Fe status [6].

Status of Fe is closely regulated to satisfy the cellular demand for iron adequately without developing toxicity from its excess. The homeostasis of this element is strongly controlled by limiting enteric iron uptake through impaired efflux from enterocytes, because the organism does not have the capacity to remove excessive amount of Fe [7].

It is suggested that iron overload may be one of diabetes risk factors. The relation between Fe and diabetes was first recognised in hereditary haemochromatosis and thalassaemia (pathological conditions), but excessive content of dietary Fe also causes the risk of diabetes. Iron takes part in β cell failure and insulin resistance, which display a direct and causal role in the pathogenesis of diabetes. Additionally, it regulates metabolism in most tissues, where adipocytes manifest a specific iron-sensing function. The molecular mechanisms mediating these effects contain modulation of adipokines and intracellular signal transduction pathways and oxidative stress [2]. The interest in the role of nutrients in diabetes is mainly concentrated on macronutrients. Iron, which is a microelement, is also tightly associated with the risk of diabetes in a many of hereditary syndromes [2]. According to Vincent, iron overload (haemochromatosis) may prevent chromium uptake by transferrin (Tf), thus leading to insulin resistance and diabetes. It is also possible that if there is excessive serum Cr level, Cr(III) binding to Tf interferes with normal iron uptake, thus affecting iron metabolism [8, 9].

On the other hand, Cr potentiates the action of insulin in patients with impaired glucose tolerance, presumably by increasing insulin receptor-mediated signalling [10, 11]. Many studies showed that Cr(III) supplementation improved insulin sensitivity and blood glucose levels in animals and humans with impaired glucose tolerance, insulin resistance and diabetes [11–14]. For these reasons, in recent years, Cr(III) supplements have become very popular therapeutics in diabetes and they have been used as agents aiding weight loss.

In 2014, the European Food Safety Authority (EFSA) released a scientific opinion document setting chromium (Cr) as a non-essential element for animals and humans [15]. According to this report, any physiological function assigned to Cr(III) is inappropriate in healthy subjects. However, chromium supplements are still very popular worldwide. Additionally, some authors reported that the risk of type 2 diabetes was lower in adults taking chromium supplements [16].

A dietary interaction between Fe(III) and Cr(III) may influence the status of these elements in the body. For this reason, the combined effects of dietary Cr3 supplementation and Fe excess on the Fe and Cr status in animal model were investigated this study.

## Materials and Methods

### Test Chemicals

Iron(III) citrate (reagent grade, 16.6% Fe) was purchased from Sigma-Aldrich, Poland. The chromium(III) complex with propionic acid (Cr3), in the form of nitrate salt [Cr_3_O(O_2_CCH_2_CH_3_)_6_(H_2_O)_3_]NO_3_, was synthesised in a laboratory at the Department of Technology and Instrumental Analysis, Poznań University of Economics, Poland, using the method described by Earnshaw et al. [17]. The Cr3 contained 21% of elemental Cr, as measured with the AAS method (spectrometer AAS-3 with BC correction, Zeiss, Germany).

### Animals and Diets

The study and all procedures were approved by the Animals Bioethics Committee of Poznań (No. 60/2013). The 36 (36♀), 6-week-old healthy female Wistar rats were used. The rats came from the Department of Toxicology, Poznań University of Medical Sciences, Poland. After 5-day adaptation to laboratory conditions, the animals were divided into six groups of approximately equal initial mean body weight of 130.5 g. During the 6-week experimental period, each of the 36 rats (6 animals/group) used in the study was individually placed in a cage and kept in a room under standard laboratory conditions (19–22 °C, 12-h light/dark cycle, 55–60% ambient air humidity). All the groups were fed semi-purified AIN-93M diets [18] (Table 1), modified according to the two-factorial experimental design.

**Table 1:** The chemical composition of experimental AIN-93 diets modified with Fe(III) and Cr(III) levels (mean ± SD)

Component	Unit	Content of compound in experimental diets					
		C1	C50	C500	H1	H50	H500
Energy	MJ 100 g^−1^	1.82 ± 0.00	1.83 ± 0.03	1.89 ± 0.06	1.88 ± 0.04	1.82 ± 0.03	1.81 ± 0.02
Fat	%	7.46 ± 0.05	7.22 ± 0.08	7.26 ± 0.31	7.31 ± 0.10	7.11 ± 0.06	6.83 ± 0.05
Protein	%	17.12 ± 0.10	17.17 ± 0.14	17.27 ± 0.24	17.42 ± 0.30	17.01 ± 0.12	17.72 ± 0.39
Carbohydrates	%	63.46	63.67	63.45	64.05	63.11	64.77
Dry mass	%	90.47 ± 0.05	90.54 ± 0.22	90.26 ± 0.08	89.99 ± 0.36	89.67 ± 0.54	89.90 ± 0.23
Ash	%	2.47 ± 0.04	2.48 ± 0.11	2.32 ± 0.50	2.36 ± 0.21	2.67 ± 0.08	2.58 ± 0.08
Ca	g kg^−1^	4.96 ± 0.13	5.16 ± 0.13	5.02 ± 0.11	5.17 ± 0.02	5.09 ± 0.11	4.94 ± 0.17
Mg	mg kg^−1^	441.42 ± 8.99	473.12 ± 1.44	511.73 ± 17.55	503.86 ± 23.80	501.62 ± 19.90	529.98 ± 15.50
Fe	mg kg^−1^	58.05 ± 0.70	57.09 ± 2.83	59.13 ± 1.98	218.12 ± 7.53	207.55 ± 21.49	229.78 ± 13.04
Zn	mg kg^−1^	52.51 ± 1.60	50.71 ± 1.90	52.41 ± 2.02	44.80 ± 4.21	40.86 ± 0.49	43.13 ± 3.00
Cu	mg kg^−1^	5.45 ± 0.94	4.43 ± 0.15	5.93 ± 0.79	5.82 ± 0.71	6.28 ± 0.20	6.62 ± 1.02
Cr	mg kg^−1^	1.24 ± 0.23	50.04 ± 6.48	425.14 ± 10.28	1.95 ± 0.61	49.42 ± 5.40	431.59 ± 14.82

The 36 healthy female Wistar rats were divided into six experimental groups (six animals in each) with different Fe levels: recommended—adequate (45 mg kg^−1^—100% RDA) and high—excessive (180 mg kg^−1^—400% RDA). At the same time, they were supplemented with Cr(III) at doses of 1, 50 and 500 mg kg^−1^ of diet, given as [Cr_3_O(O_2_CCH_2_CH_3_)_6_(H_2_O)_3_]NO_3_, also known as Cr3. The study was conducted according to the following pattern: C1—control (Fe 45 mg kg^−1^, Cr 1 mg kg^−1^); C50 (Fe 45 mg kg^−1^, Cr 50 mg kg^−1^); C500 (Fe 45 mg kg^−1^, Cr 500 mg kg^−1^); H1 (Fe 180 mg kg^−1^, Cr 1 mg kg^−1^); H50 (Fe 180 mg kg^−1^, Cr 50 mg kg^−1^); H500 (Fe 180 mg kg^−1^, Cr 500 mg kg^−1^). Female rats consumed an average of 15 g diet per day, equivalent to ~ 4 mg kg^−1^ body weight (b.w.) (C groups) and ~ 15 mg kg^−1^ b.w. (H groups) per day of Fe and 0.1; 3.3; 30 mg kg b.w. day^−1^ for Cr(III).

The rats were allowed free access to food and distilled water throughout the experiment. The feed intake was measured daily, while body weight gains were monitored weekly.

At the end of the experiment, after 12-h starvation, the rats were euthanised by asphyxiation with CO_2_. Blood was collected into tubes; tissue samples (liver, kidneys, heart, spleen, femur, ovaries, pancreas) were harvested, weighed, and frozen at − 20 °C.

### Laboratory Analyses

#### Blood Morphology

The red blood cell count (RBC) and other blood morphology indices [mean corpuscular volume (MCV), mean corpuscular haemoglobin (MCH), mean corpuscular haemoglobin concentration (MCHC), white blood cell count (WBC), platelets (PLT), lymphocytes (LYMPH), granulocytes (GRAN), minimum inhibitory dilution (MID), platelet distribution width (PDW), mean platelet volume (MPV), red cell distribution width based on standard deviation (RDW)] were measured by means of a Sysmex K-1000 haematological analyser (TAO Medical Electronics Co., Kobe, Japan). The haemoglobin (Hb) and haematocrit (HCT) were measured with an automated blood analyser (Sysmex K-1000, TAO medical Electronics Co., Kobe, Japan).

#### Blood Biochemistry

The serum iron level and total iron binding capacity (TIBC) were measured with colorimetric methods, using commercial kits (Roche Diagnostics, Germany).

Serum ferritin levels were measured using electrochemiluminescence immunoassay (ECLIA) by means of Cobas e 411 immunoassay analysers (Hitachi, Japan). The serum transferrin (Tf) levels were measured with the ELISA method (kit, DRG Pharmaceuticals, GmbH, Germany).

#### Mineral Analysis

The iron and chromium content in the diets and samples of tissues were measured with the AAS method, using AAS-3 (with BC, Carl-Zeiss, Germany) and AAS-5 EA (with BC, Jenoptic, Germany) spectrometers, after prior digestion in a Microwave Digestion System (MARS-5, CEM, USA).

The tissues (liver, kidney, spleen, heart and femur) (0.5–1.5 g) were digested with 5 ml concentrated 65% spectra pure HNO_3_ (Merck) in Teflon pressure vessels. Thereafter, the Fe concentration in the mineral solution was measured with the flame AAS method (F-AAS), while the Cr concentration was measured using a graphite furnace atomic absorption spectrometer GF-AAS (with BC, Jenoptic, Germany), having diluted the samples to the measuring range with deionised water. The accuracy of determination of Fe was assured by simultaneous analysis of the certified reference material (Pig Kidney BCR® No. 186, Brussels) while analysis Cr was assured using the certified reference material mussel tissue ERM®- CE278 (ERM). The recovery for Fe and Cr were 98 and 101%, respectively (expressed of the percentage of the mean certified values).

### Statistical Analyses

The data were analysed using two-way analysis of variance (two-way ANOVA/MANOVA, factors of Fe and Cr dietary levels, test F). If the two-way ANOVA indicated a significant Fe(III) level effect, Cr supplementation effect, or Fe x Cr interaction, a subsequent one-way ANOVA and post hoc comparison were performed using Tukey’s test. The differences were considered to be significant if the *p* values were less than 0.05. Statistical tests were performed using Statistica version 12.0 for Windows (StatSoft, Poland). The results were presented as mean ± SD.

## Results

The Fe(III) excess decreased the Cr serum concentration and the Cr liver and kidney content in the female rats (Table 2). As expected, supplementary Cr3 increased the serum Cr concentration as well as the liver and kidney Cr levels, but only the dose of 500 mg kg^−1^ had a significant effect.

**Table 2:** The main and interaction effects of different Fe(III) levels in the diet and Cr(III) supplementation on tissular contents of these elements in rats

Parameters	Unit	Main effect				Interaction effect A x B
		Factor B		Factor A		
		Cr level in diet (mg kg^−1^ diet)	Cr level in diet (mg kg^−1^ diet) 1 vs. 50 vs. 500	Fe level in diet (mg kg^−1^ diet)	Fe level in diet (mg kg^−1^ diet) 45 vs. 180	Interaction Fe x Cr
Serum Cr concentration	(ng mL^−1^)	1	15.705 ± 3.029^a^	45	33.341 ± 20.641^B^	
		50	18.629 ± 4.281^a^	180	26.974 ± 18.821^A^	NS
		500	56.139 ± 10.113^b^			
Liver Cr content	(μg g^−1^ d.m.)	1	1.398 ± 0.338^a^	45	3.010 ± 2.358^B^	
		50	1.367 ± 0.296^a^	180	2.654 ± 1.919^A^	*p* < 0.05
		500	5.603 ± 0.953^b^			
Kidney Cr content	(μg g^−1^ d.m.)	1	0.917 ± 0.123^a^	45	8.351 ± 1.112^B^	
		50	2.668 ± 0.736^a^	180	6.877 ± 7.008^A^	*p* < 0.05
		500	18.489 ± 5.327^b^			
Serum Fe concentration	(μg dL^−1^)	1	363.7 ± 43.0	45	384.9 ± 70.4	
		50	362.2 ± 66.8	180	362.0 ± 46.1	NS
		500	394.5 ± 68.6			
Liver Fe content	(μg g^−1^ d.m.)	1	843.4 ± 190.2	45	705.8 ± 75.9^A^	
		50	752.0 ± 164.1	180	963.9 ± 147.3^B^	NS
		500	850.5 ± 169.0			
Kidney Fe content	(μg g^−1^ d.m.)	1	247.0 ± 24.6	45	212.1 ± 35.6^A^	
		50	227.7 ± 68.5	180	275.0 ± 50.2^B^	*p* < 0.05
		500	241.4 ± 65.5			
Spleen Fe content	(μg g^−1^ d.m.)	1	4209.9 ± 1114.0	45	3833.6 ± 674.5	
		50	3831.9 ± 653.1	180	3822.0 ± 1167.8	NS
		500	3334.8 ± 610.0			
Heart Fe content	(μg g^−1^ d.m.)	1	273.4 ± 31.4	45	272.9 ± 35.4	
		50	280.3 ± 34.4	180	284.1 ± 25.1	NS
		500	280.8 ± 32.1			
Femur Fe content	(μg g^−1^ d.m.)	1	111.3 ± 18.0	45	108.0 ± 10.2	
		50	112.6 ± 6.8	180	118.8 ± 15.8	NS
		500	114.6 ± 14.2			

The values which do not share the same superscript letter differ in significance (two-way analysis of variance, *p* < 0.05)

*d.m* dry mass, *NS* not statistically significant

There were interaction effects of the experimental factors on the hepatic and renal Cr levels. Supplementary Cr3 increased the liver and kidney Cr content, but the oversupply of Fe(III) in the diet reduced that influence (Table 3).

**Table 3:** The combined effects of Fe(III) excess and Cr(III) supplementation on the Cr content in the tissues of female rats (mean ± SD)

Parameters	Unit	Experimental groups					
		C1 (control)	C50	C500	H1	H50	H500
		Cr status					
Serum Cr concentration	(ng mL^−1^)	16.920 ± 2.276	22.195 ± 2.554	60.908 ± 7.228	14.490 ± 3.385	15.062 ± 1.805	51.370 ± 10.871
Liver Cr content	(μg g^−1^ d.m.)	1.561 ± 0.376^a^	1.425 ± 0.246^a^	6.360 ± 0.456^c^	1.195 ± 0.131^a^	1.310 ± 0.353^a^	4.972 ± 0.774^b^
Kidney Cr content	(μg g^−1^ d.m.)	0.873 ± 0.095^a^	2.851 ± 0.295^a^	22.429 ± 4.509^c^	0.972 ± 0.145^a^	2.485 ± 1.013^a^	15.205 ± 3.469^b^
		Fe status					
Serum Fe concentration	(μg dL^−1^)	373.3 ± 50.3	346.0 ± 93.8	435.3 ± 47.3	354.0 ± 42.5	378.3 ± 39.7	353.7 ± 67.3
Liver Fe content	(μg g^−1^ d.m.)	717.1 ± 73.56	645.8 ± 75.9	750.1 ± 101.6	1001.2 ± 171.8	893.6 ± 139.6	984.5 ± 151.8
Kidney Fe content	(μg g^−1^ d.m.)	246.7 ± 20.6^abc^	175.4 ± 20.6^a^	205.5 ± 15.4^ab^	247.4 ± 32.3^abc^	297.4 ± 26.5^c^	289.342 ± 80.5^bc^
Spleen Fe content	(μg g^−1^ d.m.)	3899.4 ± 852.8	3901.6 ± 697.0	3683.4 ± 566.6	4598.1 ± 1406.4	3738.9 ± 726.7	2870.2 ± 260.1
Heart Fe content	(μg g^−1^ d.m.)	266.6 ± 29.1	286.0 ± 47.4	267.4 ± 36.3	281.7 ± 36.6	272.6 ± 4.7	298.7 ± 16.7
Femur Fe content	(μg g^−1^ d.m.)	107.5 ± 15.3	111.5 ± 3.3	105.2 ± 7.8	116.2 ± 22.2	114.0 ± 10.9	127.0 ± 10.5

Different superscript letters indicate statistically significant differences at *p* < 0.05

*d.m.* dry mass

The excessive Fe level in the diet increased the Fe content in the liver and kidneys, but supplementary Cr3 did not affect these parameters (Table 2). The interaction between dietary Fe(III) and Cr(III) levels affected the kidney Fe content. Supplementary Cr3 decreased the renal Fe level in the groups with the recommended Fe content in the diet. However, the Fe kidney level increased with the higher Cr level in the diet in the groups with the high Fe content.

Neither the Fe(III) level in the diet nor supplementary Cr3 affected the Fe content in the spleen, heart, femur and serum Fe concentration in the rats (Table 2). There were no interaction effects of the experimental factors on these indices (Table 3). However, dietary Cr supplementation tended to decrease the splenic Fe level with the Fe excess.

Table 5 shows the combined effects of excessive Fe(III) and supplementary Cr(III) on the total iron-binding capacity (TIBC), transferrin and ferritin concentration in the female rats. Different Fe(III) levels in the diet, supplementary Cr3, or their interaction did not cause differences in the TIBC, transferrin and ferritin concentration in the rats’ serum (Tables 4 and 5).

**Table 4:** The main and interaction effects of different Fe(III) levels in the diet and Cr(III) supplementation on selected biochemical and haematological indices in female rats (mean ± SD)

Parameters	Unit	Main effect				Interaction effect A × B
		Factor B		Factor A		
		Cr level in diet (mg kg^−1^ diet)	Cr level in diet (mg kg^−1^ diet) 1 vs. 50 vs. 500	Fe level in diet (mg kg^−1^ diet)	Fe level in diet (mg kg^−1^ diet) 45 vs. 180	Interaction Fe × Cr
TIBC	(μg dL^−1^)	1	457.8 ± 50.3	45	478.4 ± 45.6	
		50	479.3 ± 41.9	180	456.6 ± 40.6	NS
		500	465.3 ± 42.8			
Transferrin	(g L^−1^)	1	1.403 ± 0.147	45	1.456 ± 0.144	
		50	1.482 ± 0.134	180	1.418 ± 0.106	NS
		500	1.425 ± 0.096			
Ferritin	(ng mL^−1^)	1	2.900 ± 2.139	45	4.311 ± 2.403	
		50	5.100 ± 2.321	180	5.111 ± 3.183	NS
		500	6.133 ± 3.086			
Haemoglobin (Hb)	(g dL^−1^)	1	14.85 ± 0.40	45	15.31 ± 0.62	
		50	15.57 ± 0.47	180	15.28 ± 0.52	NS
		500	15.47 ± 0.55			
Haematocrit (HCT)	(%)	1	42.9 ± 1.7	45	44.2 ± 1.7	
		50	45.0 ± 2.0	180	44.0 ± 2.0	NS
		500	44.4 ± 1.1			
RBC	(10^6^ uL^−1^)	1	7.668 ± 0.261	45	7.904 ± 0.235	
		50	7.960 ± 0.356	180	7.746 ± 0.314	NS
		500	7.847 ± 0.149			
MCV	(fl)	1	55.9 ± 0.9	45	55.9 ± 0.9	
		50	56.6 ± 1.4	180	56.8 ± 1.2	NS
		500	56.6 ± 1.2			
MCH	(pg)	1	19.4 ± 0.4	45	19.4 ± 0.54	
		50	19.5 ± 0.7	180	19.7 ± 0.51	NS
		500	19.8 ± 0.5			
MCHC	(g dL^−1^)	1	34.68 ± 0.92	45	34.73 ± 0.89	
		50	34.60 ± 0.94	180	34.73 ± 0.63	NS
		500	34.92 ± 0.37			

The values which do not share the same superscript letter differ in significance (two-way analysis of variance, *p* < 0.05)

*NS* not statistically significant

**Table 5:** The combined effects of Fe(III) excess and Cr(III) supplementation on selected biochemical and haematological indices in female rats (mean ± SD)

Parameters	Unit	Experimental groups					
		C1 (control)	C50	C500	H1	H50	H500
		Biochemical indices					
TIBC	(ug dL^−1^)	481.7 ± 56.2	469.7 ± 58.2	484.0 ± 39.8	434.0 ± 38.1	489.0 ± 29.9	446.7 ± 44.0
Transferrin	(g L^−1^)	1.443 ± 0.206	1.460 ± 0.182	1.463 ± 0.086	1.363 ± 0.083	1.503 ± 0.101	1.387 ± 0.106
Ferritin	(ng mL^−1^)	2.100 ± 1.320	6.033 ± 2.290	4.800 ± 1.992	3.700 ± 2.787	4.167 ± 2.369	7.467 ± 3.808
		Haematological indices					
Haemoglobin (Hb)	(g dL^−1^)	14.93 ± 0.40	15.60 ± 0.62	15.40 ± 0.80	14.77 ± 0.47	15.53 ± 0.40	15.53 ± 0.32
Haematocrit (HCT)	(%)	43.20 ± 2.17	45.00 ± 1.56	44.27 ± 1.50	42.53 ± 1.43	45.00 ± 2.68	44.50 ± 0.96
RBC	(10^6^ uL^−1^)	7.780 ± 0.255	8.107 ± 0.206	7.827 ± 0.140	7.557 ± 0.261	7.813 ± 0.457	7.867 ± 0.186
MCV	(fl)	55.50 ± 0.99	55.67 ± 0.64	56.53 ± 0.93	56.3 ± 0.8	57.6 ± 1.2	56.6 ± 1.7
MCH	(pg)	19.23 ± 0.50	19.23 ± 0.55	19.77 ± 0.57	19.57 ± 0.32	19.90 ± 0.70	19.77 ± 0.61
MCHC	(g dL^−1^)	34.63 ± 1.44	34.63 ± 0.83	34.93 ± 0.57	34.73 ± 0.15	34.57 ± 1.22	34.90 ± 0.00

The values which do not share the same superscript letter differ in significance (two-way analysis of variance, *p* < 0.05)

The combined effect of the Fe(III) excess and Cr3 supplementation on the Fe metabolism was assessed on the basis of morphological and haematological blood indices, such as haemoglobin concentration (Hb), haematocrit ratio (HCT), the number of erythrocytes in the blood (RBC), mean platelet volume (MPV), mean corpuscular volume (MCV) and mean corpuscular haemoglobin concentration (MCHC) (Tables 4 and 5). The Fe(III) excess, Cr3 supplementation, or their interaction did not cause differences in the rats’ Hb, HCT, RBC, MCV, MCH and MCHC.

## Discussion

Both iron deficiency and iron excess have some consequences. Iron deficiency leads to anaemia. Up to a billion individuals worldwide suffer adverse effects from insufficient iron supply, making iron-deficiency anaemia the most common nutritional anaemia.

Lately, the health effects of iron excess have received increased attention. High amounts of Fe stored in the body cause the risk of chronic diseases, e.g. diabetes mellitus, cancer, and cardiac failure in patients with haemochromatosis [3].

Inborn iron metabolism disorders (e.g. haemochromatosis) or other factors (secondary or acquired iron overload) lead to progressive body iron overload. When the plasma Fe content exceeds the iron-binding capacity of transferrin, Fe accumulates in the body and causes cell damage with different clinical symptoms, such as inflammation, arrhythmias and diabetes mellitus [3].

The beneficial or harmful effect of elements depends, to a degree, on the influence of external factors, e.g. nutrition, and internal factors such as individual absorption and metabolism of these elements, age, gender and genetic disposition. Moreover, the effect of trace elements depends not only on their quantity in the diet but also on their interaction.

The bioavailability of Fe is more important than its absolute levels. Despite the extensive regulation of the iron uptake, dietary Fe excess may cause higher tissue Fe levels than are necessary to maintain normal erythropoiesis and metabolic function [2].

The degree of iron absorption varies with food. Apart from the degree of organism saturation with this nutrient, the type of product consumed, the physical and chemical form of iron, and interactions between dietary components are also important [19, 20]. Two types of Fe are found in food: heme Fe(II)—in products of animal origin, and non-heme Fe(III)—mainly in products of plant origin (cereal products, dry seeds of legumes and in some leafy vegetables). The relatively low bioavailability of Fe from cereal grains and legumes is attributed to the content of phytic acid in these foods. The bioavailability of heme iron may be as high as 23–27%, while the bioavailability of non-heme iron ranges from 1 to 8% [20].

Many variables need to be considered to identify the optimal range of iron intake, such as physiological needs and factors that enhance or inhibit the absorption of iron from foods, once absorbed dietary iron is not actively excreted. Therefore, under most conditions, the absorption of dietary Fe, whether from foods or supplements, is the critical determinant of the iron status. Iron absorption is influenced by a number of factors, but feedback regulation and the bioavailability of iron-containing foods are two key considerations [4].

High Fe intake is most likely the result of dietary supplementation and the consumption of foods fortified or enriched with iron. The results of some studies support the suggestion that the absorption of Fe from supplements may be inhibited by other mineral ingredients [1]. If Fe absorption is tightly regulated through feedback, it is unlikely that modest levels of supplemental iron pose a health risk to otherwise healthy adults [1].

There are close relations between the metabolisms of different trace elements, including iron, based on antagonistic or synergistic interactions [21]. Interactions of different trace elements with Fe determine the relationship between changes in the trace element status in the organism. Higher content of trace elements antagonistic to Fe, such as Co, Zn, Cu, Cr, and Ca, which impair Fe absorption or its physiological impact, can disturb the metabolism of this element and vice versa [21]. Some data suggest that Fe and Cr may compete for transferrin [8, 9, 22].

A number of studies shown that different chemical forms of Cr(III) have diverse rates of absorption. It is observed that inorganic Cr(III), e.g. CrCl_3_, is very poorly absorbed (0,5–1%) than organic (2–5%), which has greater bioavailability [23]. A variety of organic forms of Cr are now available worldwide. One of them is chromium(III) propionate (aka Cr3). As with all minerals, different forms would be expected to have different bioavailability [23]. The relative bioavailability of different forms will affect the ultimate tissue supply of the mineral, and consequently, it will influence both the potential biological response(s) and metabolism.

Studies have proved that some Cr supplements, like the tricentric Cr(III) complex with propionic acid (Cr3), are characterised by much higher absorption, i.e. 40–60% [24, 25]. Lindemann et al. [23] studied relative bioavailability in response to high-level, short supplementation among four organic Cr sources as the Cr concentration in several tissues. They found that Cr affected multiple tissues, which is conclusive evidence of absorption and deposition. Not all forms affected the tissue Cr concentration equally. The results showed obvious differences in the tissue content of organic Cr from different sources. The mean bioavailability relative to chromium tripicolinate (CrTP) across the tissues amounted to 13.1% for chromium propionate (CrPrp; 0.4–26.8%), 50.5% for chromium methionine (CrMet; 36.2–79.1%), and 22.8% for Cr yeast (CrY; 2.5–47.9%). Recently, Cr has been found to be a beneficial rather than essential trace element for mammals and has gained popularity as a nutritional supplement. Cr supplements are commonly used for treating diabetes and obesity despite ambiguous reports on their efficacy [14].

Yoshida et al. [26] showed that the increase in the liver, kidney and femur Cr content depended on the dietary Cr(III) level. They found that the difference in the chemical species of supplemented Cr did not influence the liver or kidneys, but CrCl_3_ caused significantly higher Cr accumulation than CrPic in the femur of Wistar rats given 100 μg Cr g^−1^. Moreover, the daily urinary Cr excretion increased along with the dietary Cr(III) level. Rats given CrPic had significantly higher urinary Cr excretion than those given CrCl_3_. This finding partly corresponds to our results. In our previous study, we noted that the high dietary Cr3 doses (100–1000 mg kg^−1^ of diet) increased the content of this element in female rats’ tissues, depending on the dosage [27]. Other authors found similar relationships between the intake of Cr(III) and its concentration in the liver and kidney tissues in normal rats [25, 28, 29] as well as rats with diabetes mellitus type 1 and 2 [30, 31], in quails [32], chickens [33], pigs [34] and lambs [35]. Also, Wang et al. [36] noted that the accumulation of Cr in finishing pigs’ tissue increased after supplementation with different forms of Cr (CrCl_3_, CrPic, CrNano) at a dose of 200 μg kg^−1^ for 40 days. Li et al. [37] made the same observations.

Researchers observed that absorbed Cr was mostly excreted in the urine, which might be the reason for the highest Cr residue in the kidneys [36, 38]. Dallago et al. [35] observed a positive linear relationship between the dose administered and the accumulation of Cr in the heart, lungs and testis. Urinary Cr excretion occurred in a time- and dose-dependent manner, so the longer or the more dietary Cr was provided, the greater the excretion of the element was in lambs supplemented with dietary CrPic.

Liu et al. [33] demonstrated that long-term (42 days) exposure of chickens to high Cr(III) doses via drinking water significantly increased the Cr content in the serum and in the brain. Moreover, the authors noted that the Fe and Cu contents increased in the serum, but the contents of these elements decreased in the brain. The research showed that the blood-brain barrier may prevent the accumulation of these elements in the brain exposed to CrCl_3_.

Supplementary Cr3 (10 and 100 mg Cr kg^−1^ b.w.) given for 4 weeks decreased the serum Fe concentration and the Fe content in the liver and kidneys of healthy female Wistar rats [27]. Our previous study showed that the blood morphological indices remained unchanged after supplementation with Cr3 at doses of 100–1000 mg kg^−1^, which means that Cr3 did not affect erythropoiesis [39]. The serum, hepatic and renal Fe concentrations were lower but these changes occurred only after the application of very high doses of Cr(III) (50 and 100 mg Cr(III) kg^−1^ b.w.) [27].

The exposure to high Fe(III) level increased the Fe content in all the tissues examined, especially in the liver and kidneys, but decreased the serum, hepatic and renal Cr concentrations. However, it did not significantly change the TIBC, transferrin and ferritin concentrations in the serum as well as most of the haematological parameters. Supplementary Cr3 did not change most of the indices of Fe metabolism, except the Fe kidney concentration. It was found that supplementary Cr3 decreased the renal Fe level in the groups with the adequate Fe content in the diet. Nevertheless, in the groups with the oversupply Fe in the diet, the Fe kidney content increased with the higher dietary Cr level. There were no significant changes in most biochemical and haematological parameters after Cr3 supplementation. There was a beneficial effect of Cr(III) with excessive Fe. The high amount of Fe in the diet might inhibit Cr absorption in the small intestine. Under normal conditions, only about 30% of the potential Fe(III) binding sites in Tf are occupied, leaving the unoccupied binding sites to potentially bind other metal ions [8, 9, 40, 41]. When the saturation of transferrin with iron rises to over 50%, iron competes with chromium binding, affecting its transport [42]. The minimal effect of the Fe excess (400% RDA) on the metabolic rates of this element may be caused by the fact that Fe(III) is poorly absorbed.

Yasutake and Hirayama [43] reported that the addition of 3.5% Fe(II) fumarate to the diet caused enhanced oxidative status in the liver and kidney, manifested by increased TBARS levels, as well as tissue Fe accumulations in male Wistar rats.

Some researchers have found lower Cr concentrations in the blood of anaemic patients than in healthy subjects [21, 44]. We also observed that supplementary Cr3 decreased the kidney Fe content, but in Fe-deficient rats, the impact was weaker than in those with an adequate Fe level in the diet (data not published yet). Deficiencies of trace elements synergistic to iron, such as copper, chromium, nickel, sodium, and potassium, implicated in iron metabolism or processes of haematopoiesis, can substantially contribute to the aetiology of iron-deficiency anaemia (IDA) [21].

On the contrary, Anderson et al. [45] observed a reduce Fe content in the tissues of rats supplemented with chromium(III) chloride. Similarly, the results obtained by Ani and Moshtaghie [46] reported that decreased transferrin saturation and tissue stores of Fe as well as reduce haemoglobin and haematocrit indices in animals fed a diet with a high content of Cr(III). This was an antagonistic competition between trivalent chromium and trivalent iron for binding to apotransferrin [8, 9]. Although, Sun et al. [32] reported that long-term (24 weeks) Cr3 administered to healthy Sprague-Dawley (SD) rats at a dose of 20 μg kg^−1^ b.w. did not cause significant changes in content of Fe in the liver and kidney. Likewise, Love et al. [46] found that the supplementary Cr(III) (16–2000 μg kg^−1^ diet) given for 23 weeks had no effect on the blood iron concentration in ZKL rats. No adverse effects of Cr3 on the Fe status when given to normal and with type 2 diabetes rats for 24 weeks at doses of 250–1000 μg of Cr kg^−1^ b.w. were observed [24]. A 90-day sub-chronic toxicity study and long-term treatment (52 weeks) with complex Cr(III) with niacin reported not affect the parameters of Fe metabolism in rats [47, 48]. Other studies [49] demonstrated that addition of Cr(III) in a diet enriched with cellulose and/or pectin increased the Cr and Fe levels in the femurs but did not affect the hepatic, renal and muscular Fe contents of male rats.

Studies on humans showed that CrPic supplementation at a dose of 200 μg increased the serum Cr concentration and urinary Cr excretion but did not affect measurements of the iron status in premenopausal women [50]. Lukaski et al. [51] suggested the adverse effect of high-dose and long-term chromium supplementation on iron metabolism and status in adults.

Our previous study showed that Cr3 supplementation (7.2 mg Cr(III) kg^−1^ body weight per day) of rats for 21 days of gestation increased the dams’ liver and kidney Cr levels but did not affect maternal Fe levels or foetal Cr and Fe levels [52]. Bourque et al. [53] noticed that maternal iron intake impacts the metabolic programming in their offspring. However, the phenotype depends on the period of altered iron status and the direction of change (iron deficiency or excess). In rodents, prenatal Fe deficiency increased susceptibility to high-fat-diet-induced glucose intolerance, hypertension, and obesity [53]. The effects of Fe on metabolic programming might be diet-dependent, what is in agreement with its effects on mediators (e.g. AMPK). So far, studies on humans mostly focused on iron deficiency rather than excess, and not in the context of the risk of diabetes. The Fe level in diet may be a factor signalling the fed versus fasted state of the body, which could show epigenetic modifications initiating an altered metabolic status that is an agreement with nutritional availability. The further research to evaluate the role of prenatal iron exposure in adult glucose homeostasis are required. Both Fe and Cr may be important in the pathogenesis of diabetes, but the direction of their action may be opposite. It suggests the Cr deficiency as a possible risk factor in the development of diabetes mellitus. Basaki et al. [10] showed that patients with diabetes mellitus had lower Cr, Zn and Cu concentrations than healthy subjects; however, the difference in the Fe concentration was not significant. The Cr(III) treatment is helpful for glycaemic control in diabetic patients. On the other hand, the results obtained by Herring et al. [54] suggest that long-term (15 months) Cr3 supplementation does not significantly impact metabolic responses in blood glucose concentration to glucose and insulin in male rats’ intake of a high-fat diet, high-carbohydrate cafeteria-style diet or normal diet. By contrast, Cr3 supplementation at doses of 10 and 50 mg kg^−1^ diet for 8 weeks increased the kidney Fe content in rats fed a high-fat diet [55].

Iron overload (haemochromatosis) may prevent chromium uptake by Tf, thus leading to insulin resistance and diabetes. It is suggested that excess Fe(III) lower the ability of Cr(III) to bind to Tf, when binding of Fe(III) to transferrin (at physiological concentrations) is not influenced by a physiologically relevant content of Cr(III) [8, 56].

Inversely, if there is chromium excess in the serum, Cr(III) binding to Tf may interfere with normal iron uptake, thus affecting iron metabolism [8, 9, 56]. Furthermore, Fe overload increases oxidative stress [57]. The evidence that Fe overload could contribute to abnormal glucose metabolism was first derived from the observation that the incidence of diabetes increased in hereditary haemochromatosis [57, 58]. In an animal model of haemochromatosis, iron excess and oxidative stress mediate apoptosis of pancreatic islets with a resultant decrease in insulin secretory capacity [59]. The exact mechanism of iron-induced diabetes is uncertain. Patients with unexplained hepatic iron overload were found to be insulin resistant, which suggests a common etiological link between hepatic iron, hepatic dysfunction and insulin resistance [57].

Iron excess has been linked to oxidative damage to DNA, lipids and proteins, which has been implicated in diabetes, cardiovascular disease, atherosclerosis and neurological degeneration (e.g. in Alzheimer’s disease) or other health consequences [2].

## Conclusions

This study showed that exposure to high Fe(III) level alone or in combination with Cr caused Fe accumulation in tissues, especially in the liver and kidneys, but did not significantly change the TIBC, transferrin, ferritin concentration in the serum and most haematological parameters. Moreover, the Cr concentrations in the serum, liver and kidneys decreased. On the other hand, the results showed that Cr(III) dosed individually or in combination with a high level of Fe(III) disturbed the Cr content in the liver and kidneys of healthy female rats. However, it did not change most of the parameters of Fe metabolism, except the Fe kidney concentration.

The research findings showed a relationship between Fe(III) and Cr(III) metabolism in healthy female rats. However, the direction of change varied and depended on relative amounts of these elements in the diet. Further studies are required to set the biological interaction between Fe and Cr under various exposure regimens and elements proportions.
